# *Pseudomonasextremorientalis* BU118: a new salt-tolerant laccase-secreting bacterium with biotechnological potential in textile azo dye decolourization

**DOI:** 10.1007/s13205-016-0425-7

**Published:** 2016-04-18

**Authors:** Mohamed Neifar, Habib Chouchane, Mouna Mahjoubi, Atef Jaouani, Ameur Cherif

**Affiliations:** 1 BVBGR-LR11ES31, ISBST, University of Manouba, Biotechpole Sidi Thabet, 2020 Ariana, Tunisia; 2 MBA-LR03ES03, FST, University of Tunis El Manar, Campus Universitaire, 2092 Tunis, Tunisia

**Keywords:** *Pseudomonas extremorientalis* laccase, Azo dye decolourization, Salt tolerance, Response surface methodology, Central composite design

## Abstract

The present investigation focused on screening of a new potent strain for laccase production and optimizing the process parameters to achieve the maximum enzymatic decolourization of textile azo dye Congo red. Seven hydrocarbonoclastic bacterial strains were selected as positive in laccase production in solid medium using 2,6 dimethoxyphenol as an enzyme activity indicator. The best enzyme producer *Pseudomonas* *extremorientalis* BU118 showed a maximum laccase activity of about 7000 U/L of wheat bran under solid-state conditions. The influence of different concentrations of dye, enzyme, salt and various incubation times on Congo red decolourization was studied using response surface methodology to find the optimum conditions required for maximum decolourization by *P.* *extremorientalis* laccase. The enzyme exhibited a remarkable colour removal capability over a wide range of dye and salt concentrations. The above results show the potential use of this bacterial laccase in the biological treatment of the textile effluent.

## Introduction

Laccase (benzenediol:oxygen oxidoreductase, EC 1.10.3.2) is one of the best-known multicopper enzymes and catalyzes the oxidation of a variety of aromatic compounds, in particular phenolic substrates, coupled to the reduction of molecular oxygen to water. These enzymes are of low substrate specificity and oxidize a broad group of monophenols, diphenols, polyphenols and methoxy-substituted phenols as well as aromatic amines and metallic ions (Claus 2003; Canas and Camarero 2010; Neifar et al. 2011). These enzymes have received particular interest in bioremediation applications of treating coloured industrial wastewaters, because they are biodegradable, cost-effective and environmentally friendly (Benzina et al. 2012; Neifar et al. 2015; Singh et al. 2015).

Laccases from plant and fungal sources, especially white-rot fungi, have been studied extensively (Arora and Sharma 2010; Skariyachan et al. 2016). However, laccases have been discovered in a small number of bacteria including *Bacillus* *subtilis*, *Bordetella* *campestris*, *Caulobacter* *crescentus*, *Escherichia* *coli*, *Mycobacterium* *tuberculosis*, *Pseudomonas* *syringae*, *P.* *aeruginosa*, *P.* *putida*, *P.* *fluorescens*, *Yersinia* *pestis* and *Stenotrophomonas* *maltophilia* (Claus 2003; Sharma et al. 2007; Imran et al. 2012; Kuddus et al. 2013; Vandana and Peter 2014; Verma et al. 2016). These bacterial laccases have the ability to perform the activity at crucial conditions like in the presence of high salt concentrations and even at alkaline pH values (Margot et al. 2013). Although some bacterial laccases have been well characterized, little information is available concerning their substrate specificities towards colour removal (Hadibarata and Tachibana 2009).

High amounts of chemically different dyes are used for the textile industry and a significant amount of these dyes enter the environment as coloured wastewaters. Not all the dyes currently used could be removed with physical–chemical treatments and sometimes the degradation products are more toxic (Shah et al. 2013; Lalnunhlimi and Krishnaswamy 2016). Currently, one of the possible alternatives for the treatment of textile wastewaters is the use of microbial laccases, which can oxidize a wide spectrum of synthetic recalcitrant dyes (Couto and Toca-Herrera 2006; Daassi et al. 2013; Hafshejani et al. 2014; Neifar et al. 2015).

With this in view, the present investigation describes a new laccase-producing bacterium with biotechnological potential in dye decolourization. The specific aims of this study were (1) to select the best laccase-producing bacterium from hydrocarbonoclastic bacteria collection screened previously for their abilities to degrade hydrocarbons and produce biosurfactants (Mahjoubi et al. 2013); (2) to produce laccase under solid-state fermentation and finally (3) to optimize the decolourization of the recalcitrant azo dye Congo red under saline conditions by response surface methodology (RSM).

## Materials and methods

### Screening and phylogenetic analysis of laccase-producing bacteria

Hydrocarbonoclastic bacterial strains isolated from contaminated sediments from a refinery harbour of the Bizerte coast in Northern Tunisia, and affiliated to *Achromobacter* *xylosoxidans* BU22 (KC153020), *Acinetobacter* *venetianus* BU19 (KC152985), *Acinetobacter* *beijerinckii* BU45 (KC152987), *Luteibacter* *rhizovicinus* BU33 (KC152978), *Gordonia* *amicalis* BU147 (KC153019), *Ochrobactrum* *grignonense* BU72 (KC153015) and *P.* *extremorientalis* BU118 (KC153004) (Mahjoubi et al. 2013), were subjected to plate test screening method (Kiiskinen et al. 2004). The assay plate contained 15 ml of tryptic soy agar (TSA; Difco) medium amended with 0.01 % of 2,6 dimethoxyphenol (DMP) to detect laccase activity (YunYang et al. 2008). The pH was adjusted to 7.5 before autoclaving at 121 °C for 15 min. The plates were incubated at 30 °C for 5–6 days. The presence of brick colour around the colonies was considered as DMP-oxidizing laccase-secreting organism.

16S rRNA gene sequence of the best enzyme-producing strain (BU118) was compared with sequences available in the nucleotide database using the BLAST algorithm at the National Center for Biotechnology Information (NCBI) database (http://www.ncbi.nlm.nih.gov) (Mahjoubi et al. 2013). The phylogenetic tree representing the position of BU118 relative to the closest related type strains of the other species within the genus *Pseudomonas* available at the NCBI database was constructed by the neighbour-joining method (Saitou and Nei 1987) using MEGA software version 6.06 (Tamura et al. 2013). The confidence values of branches of the phylogenetic tree were determined using bootstrap analysis based on 1000 resamplings (Felsenstein 1985).

### Laccase production by solid-state fermentation and extraction of crude enzyme

Wheat bran procured from the local market was used as solid substrate for the production of laccase by the selected hypersecretory strain. Five grams of substrate was transferred into 100 ml conical flasks and then moistened with 10 ml of tryptic soy broth (TSB; Difco) medium (Mahjoubi et al. 2013). The initial pH of the medium was set at 8. All preparations in the flasks were autoclaved at 121 °C for 15 min and inoculated with 0.1 ml of 1.0 % v/v (O.D. 600 nm ≈ 1.20) inoculum from the mother liquid culture. After incubation, 10 ml of 0.1 M Tris–HCl pH 8 buffer was added to the flask and stirred for 30 min for the extraction of crude laccase. Then the contents of the flask were centrifuged at 10,000×*g* for 15 min at 4 °C, and the supernatant was treated as crude enzyme.

### Enzyme assay

The laccase activity was measured by monitoring the oxidation of 5 mM DMP buffered with 50 mM phosphate (pH 8.0) at 469 nm for 1 min (Molina-Guijarro et al. 2009). To calculate the enzyme activity, an absorption coefficient of 27,500 M/cm was used. One unit of laccase activity was defined as the amount of enzyme required to oxidize 1 µM of 2,6-DMP oxidized per min (Neifar et al. 2011).

### Properties of crude laccase

The optimum pH was determined with DMP as a substrate dissolved in the following buffer systems: potassium phosphate buffer (pH 6.0–8.0) and glycine–NaOH buffer (pH 9.0–11.0). The optimum temperature was determined for the laccase at different temperatures (10–70 °C). For the study of halostability, the enzyme was pre-incubated with NaCl (0–3 M) at room temperature for 1 h and the enzyme activity was determined.

### Congo red decolourization

Dye decolourization capability of the crude laccase from *P.* *extremorientalis* was accessed using Congo red dye. The reaction mixture contained 50 mM phosphate buffer (pH 8.0), laccase, dye and salt. The reaction mixture was incubated at 30 °C in the dark and the dye decolourization was measured by monitoring the decrease in absorbance maximum of the dye (*λ*_max_ = 495 nm) in the UV/VIS scanning spectrophotometer (Shimadzu UV-1800 PC model Kyoto, Japan). Decolourization was expressed in terms of percentage and calculated as:

$${\text{Decolourization }}\left( \% \right) = \left[ {{{\left( {{\text{initial absorbance}} - {\text{observed absorbance}}} \right)} \mathord{\left/ {\vphantom {{\left( {{\text{initial absorbance}} - {\text{observed absorbance}}} \right)} {{\text{initial absorbance }} \times 100}}} \right. \kern-0pt} {{\text{initial absorbance }} \times 100}}} \right].$$

To set up a control, heat-denatured enzyme was added to the reaction mixture instead of active enzyme (Daassi et al. 2013). The decolourization yield was calculated from the difference between the decolourization produced in the reaction mixture containing the active enzyme and that containing the heat-inactivated enzyme.

### Optimization of Congo red decolourization by RSM

To optimize the Congo red decolourization by *P.* *extremorientalis* laccase, a standard RSM design called central composite design (CCD) was applied to study the decolourization reaction variables (Myers et al. 2009). This method is suitable for fitting a quadratic surface and helps to optimize the effective parameters with a minimum number of experiments, as well as to analyse the interaction between the variables (Goupy 1999; Myers et al. 2009). A CCD consisting of 30 experiments was chosen for the optimization of CR decolourization. Four independent variables, namely enzyme concentration (*X*_1_), dye concentration (*X*_2_), NaCl concentration (*X*_3_) and incubation time (*X*_4_), were evaluated at three levels (Table 1), and the percentage of CR decolourization was the dependent variable (response). The following equation was used to establish the quadratic model:

$$Y = \, \beta_{0} + \, \beta_{1} X_{1} + \, \beta_{2} X_{2} + \, \beta_{3} X_{3} + \, \beta_{4} X_{4} + \, \beta_{11} X_{1}^{2} + \, \beta_{22} X_{2}^{2} + \, \beta_{33} X_{3}^{2} + \, \beta_{44} X_{4}^{2} + \, \beta_{12} X_{1} X_{2} + \, \beta_{13} X_{1} X_{3} + \, \beta_{14} X_{1} X_{4} + \, \beta_{23} X_{2} X_{3} + \, \beta_{24} X_{2} X_{4} + \, \beta_{34} X_{3} X_{4} ,$$

where *Y* is the response (% decolourization); *X*_*i*_ and *X*_*j*_ are uncoded independent variables; and *β*_0_, *β*_*j*_, *β*_*jj*_ and *β*_*jk*_ are intercept, linear, quadratic and interaction constant coefficients, respectively.

Validation of the optimum decolourization results predicted by the model was conducted in triplicate. The generation and the data treatment of the four factors Box–Behnken experimental design were performed using the software NemrodW (Mathieu et al. 2000).

**Table 1:** Experimental conditions of the CCD design in coded and natural variables and the corresponding experimental and estimated responses

No. exp.	*X*_1_	*X*_2_	*X*_3_	*X*_4_	Enzyme (U/ml)	Dye (mg/l)	Salt (%)	Time (h)	Measured decolourization (%)	Estimated decolourization (%)
1	−1.0	−1.0	−1.0	−1.0	0.2	50.0	0.0	4.0	38.0	38.24
2	1.0	−1.0	−1.0	−1.0	1.0	50.0	0.0	4.0	44.0	44.50
3	−1.0	1.0	−1.0	−1.0	0.2	250.0	0.0	4.0	53.0	52.95
4	1.0	1.0	−1.0	−1.0	1.0	250.0	0.0	4.0	61.0	60.96
5	−1.0	−1.0	1.0	−1.0	0.2	50.0	5.0	4.0	63.0	62.95
6	1.0	−1.0	1.0	−1.0	1.0	50.0	5.0	4.0	65.0	64.96
7	−1.0	1.0	1.0	−1.0	0.2	250.0	5.0	4.0	61.0	61.41
8	1.0	1.0	1.0	−1.0	1.0	250.0	5.0	4.0	65.0	65.17
9	−1.0	−1.0	−1.0	1.0	0.2	50.0	0.0	24.0	61.0	61.06
10	1.0	−1.0	−1.0	1.0	1.0	50.0	0.0	24.0	65.0	65.07
11	−1.0	1.0	−1.0	1.0	0.2	250.0	0.0	24.0	41.0	41.52
12	1.0	1.0	−1.0	1.0	1.0	250.0	0.0	24.0	47.0	47.28
13	−1.0	−1.0	1.0	1.0	0.2	50.0	5.0	24.0	75.0	75.52
14	1.0	−1.0	1.0	1.0	1.0	50.0	5.0	24.0	75.0	75.28
15	−1.0	1.0	1.0	1.0	0.2	250.0	5.0	24.0	40.0	39.73
16	1.0	1.0	1.0	1.0	1.0	250.0	5.0	24.0	41.0	41.24
17	−1.0	0.0	0.0	0.0	0.2	150.0	2.5	14.0	51.0	49.64
18	1.0	0.0	0.0	0.0	1.0	150.0	2.5	14.0	55.0	53.52
19	0.0	−1.0	0.0	0.0	0.6	50.0	2.5	14.0	72.0	70.41
20	0.0	1.0	0.0	0.0	0.6	250.0	2.5	14.0	62.0	60.75
21	0.0	0.0	−1.0	0.0	0.6	150.0	0.0	14.0	48.0	46.41
22	0.0	0.0	1.0	0.0	0.6	150.0	5.0	14.0	57.0	55.75
23	0.0	0.0	0.0	−1.0	0.6	150.0	2.5	4.0	60.0	58.86
24	0.0	0.0	0.0	1.0	0.6	150.0	2.5	24.0	60.0	58.30
25	0.0	0.0	0.0	0.0	0.6	150.0	2.5	14.0	59.0	56.90
26	0.0	0.0	0.0	0.0	0.6	150.0	2.5	14.0	51.0	56.90
27	0.0	0.0	0.0	0.0	0.6	150.0	2.5	14.0	56.0	56.90
28	0.0	0.0	0.0	0.0	0.6	150.0	2.5	14.0	61.0	56.90
29	0.0	0.0	0.0	0.0	0.6	150.0	2.5	14.0	49.0	56.90

## Results and discussion

### Selection of the laccase-producing bacterium

**Fig. 1:**
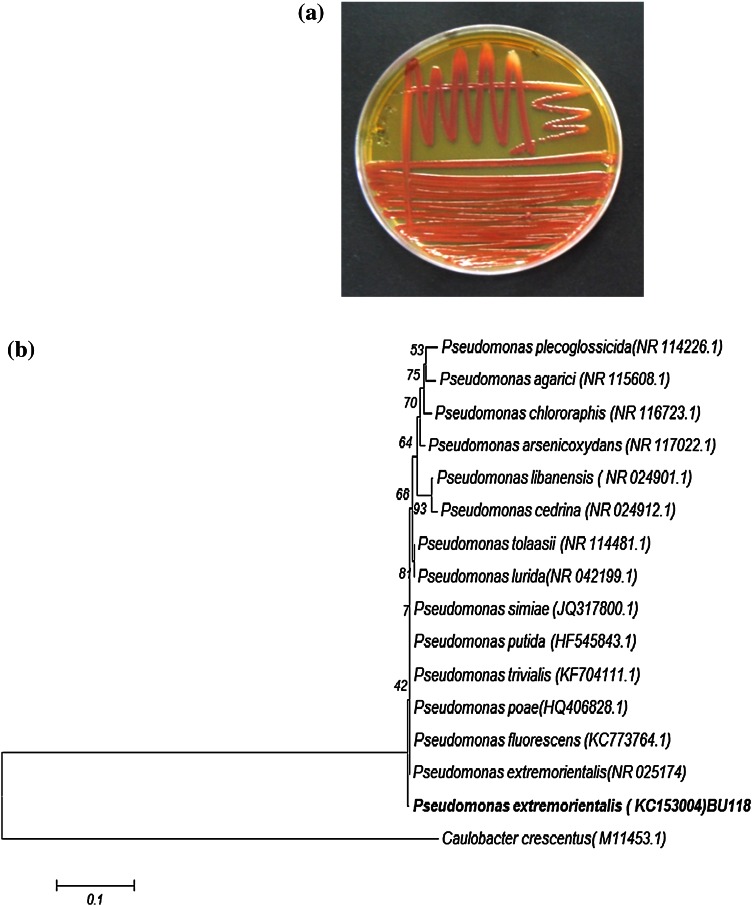
**a** Petriplate showing *Pseudomonas* *extremorientalis* BU118 grown in 2,6-dimethoxyphenol-supplemented solid medium. The production of an intense *brown colour* is considered as a positive reaction for the presence of laccase activity. **b** Phylogenetic analysis of 16S rRNA gene sequence of bacterial isolate *P.* *extremorientalis* strain BU118 based on 16S rDNA partial sequences. Phylogenetic dendrogram was evaluated by performing bootstrap analysis of 1000 data sets using MEGA 6.06 software. 16S rRNA sequence accession numbers of the reference strains are indicated in *parentheses*

Hydrocarbonoclastic bacteria previously isolated from petroleum-contaminated sediments in Tunisia (Mahjoubi et al. 2013) were screened for laccase activity on solid media containing DMP as an indicator compound (YunYang et al. 2008). The formation of brown colour around the colonies after incubation at 30 °C for 4 days indicated the presence of the laccase enzyme. The colour intensity varies due to the variability in the concentration of laccase production (Amutha and Abhijit 2015). On the basis of this screening, seven potential species belonging to six genera designated as *Achromobacter* *xylosoxidans* BU22, *Acinetobacter* *venetianus* BU19, *Acinetobacter* *beijerinckii* BU45, *Luteibacter* *rhizovicinus* BU33, *Gordonia* *amicalis* BU147, *Ochrobactrum* *grignonense* BU72 and *P.* *extremorientalis* BU118, showed positive laccase activities. Out of seven, the laccase-positive isolate, *P.* *extremorientalis* BU118, was found to be the most potential isolate producing laccase on the basis of DMP oxidation in the plate screening test (Table 2; Fig. 1a). Therefore, BU118 was selected for further investigation based on the highest enzyme activity. The phylogenetic tree obtained when the 16s RNA gene sequence of the organism was analysed is shown in Fig. 1b. Bacterial laccase producers belonging to the *Pseudomonas* species have been previously described for *P.* *putida* (McMahon et al. 2007; Kuddus et al. 2013), *P.* *fluorescens* (Vandana and Peter 2014), *P.* *aeruginosa* (Peter and Vandana 2014) and *P. desmolyticum* (Kalme et al. 2009).

**Table 2:** Colour intensity due to laccase production by different positive bacterial strains in the presence of the DMP substrate

Strain	Accession number	Closest relative	DMP oxidation and brick red colour intensity
BU22	KC153020	*Achromobacter* *xylosoxidans*	+
BU19	KC152985	*Acinetobacter* *venetianus*	+++
BU45	KC152987	*Acinetobacter* *beijerinckii*	+++
BU33	KC152978	*Luteibacter* *rhizovicinus*	++
BU147	KC153019	*Gordonia* *amicalis*	++
BU72	KC153015	*Ochrobactrum* *grignonense*	++
BU118	KC153004	*Pseudomonas* *extremorientalis*	++++

Colour intensity (++++ very good, +++ good, ++ light, + very fent)

### Production of laccase under solid-state fermentation

**Fig. 2:**
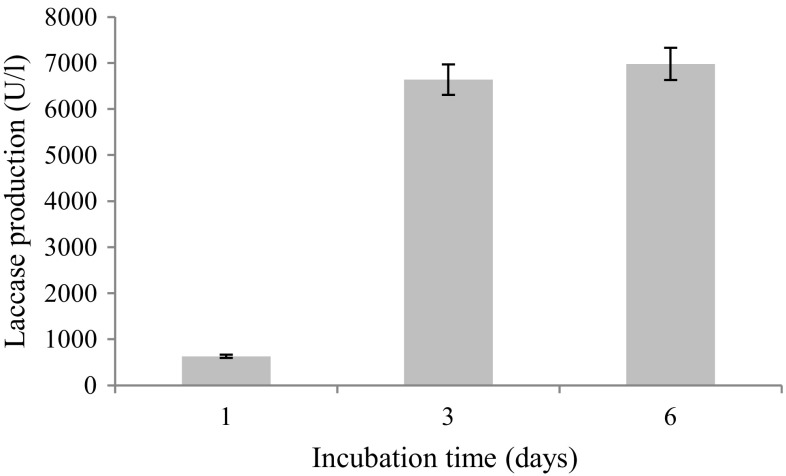
Laccase production by *Pseudomonas* *extremorientalis* BU118 grown on wheat bran-based solid medium

The production of laccase by bacteria under solid-state cultivation is found to be economical (Muthukumarasamy and Murugan 2014). As shown in Fig. 2, laccase production by *P.* *extremorientalis* first appeared on the first day (630 U/L) and increased afterwards, peaking on the 6th day (6980U/L). This result is in agreement with that of Osma et al. (2006) and El-Batal et al. (2015), who reported high microbial laccase activities on wheat bran under solid-state fermentation. The authors pointed out that the inductive laccase capability of wheat bran may be directly related to its phenolic compounds such as ferulic, coumaric and syringic acids.

### Properties of the extracellular laccase

As shown in Table 3, *P.* *extremorientalis* laccase had maximum activity at 40–50 °C, but is active over a wide range of temperature (20–70 °C). The enzyme was active in the pH range of 7.0–10.0, with an optimum at pH 8.0. In terms of stability, the enzyme retained up to 100 % of its initial activity after 24 h of incubation at pH values between 7 and 10. In contrast, the enzyme lost 100 % of its activity when incubated at acidic pH values. This activity against a phenolic compound at neutral to alkaline pH is only found in a few bacterial and fungal laccases (Machczynski et al. 2004; Ruijssenaars and Hartmans 2004; Kuddus et al. 2013; Peter and Vandana 2014; Vandana and Peter 2014). *P.* *extremorientalis* laccase showed also exceptional resistance to sodium chloride, maintaining 100 % activity at high concentrations of this salt (2 M) at pH 8; thus, it can be considered as a potentially good candidate for textile wastewater treatment. Indeed, industrial dyeing requires the neutral electrolyte NaCl up to 50 g/l, which represents one of the main obstacles for application of fungal laccases due to their inhibition by chloride ions (Jimenez-Juarez 2005; Loncar et al. 2013). Among bacterial laccases, halide tolerance has been also described in the laccases of *Marinomonas* *mediterranea* (Jimenez-Juarez 2005) and *Streptomyces* *ipomoeae* (Molina-Guijarro et al. 2009).

**Table 3:** Properties of the extracellular laccase produced by *P.* *extremorientalis* BU118

Parameter	Value
pH opt.	8.0
T opt.	40–50 °C
NaCl stability (relative activity)	
0 M	100.0 %
1 M	120.5 %
2 M	111.2 %
3 M	87.6 %

### Performance of crude laccase on azodye decolourization: optimization study

Since the enzymatic decolourization is a multivariable process, optimization of *P.* *extremorientalis* laccase-mediated Congo red decolourization was carried out using RSM (Goupy 1999; Myers et al. 2009). A CCD was chosen to determine the optimum requirement of enzyme (*X*_1_), dye (*X*_2_), salt (*X*_3_) and time (*X*_4_) for maximum dye decolourization (Table 1). The mathematical expression of the relationship to Congo red decolourization with the variables *X*_1_, *X*_2_, *X*_3_ and *X*_4_ is as follows:

$$Y = 56.903 + 1.944X_{1} - 4.833X_{2} + 4.667X_{3} - 0.278X_{4} - 5.322X_{1}^{2} + \, 8.678X_{2}^{2} - 5.822X_{3}^{2} + \, 1.678X_{4}^{2} + \, 0.438X_{1} X_{2} - \, 1.063X_{1} X_{3} - \, 4.063X_{2} X_{3} - \, 0.563X_{1} X_{4} - 8.563X_{2} X_{4} - 2.563X_{3} X_{4} .$$

ANOVA of the regression model demonstrated a high significance (*P* < 0.0001) of the model and an insignificant lack of fit (Table 4). The integrity of the model can be checked by the determination coefficient *R* ^2^ and the multiple correlation coefficient *R*. It measures the proportion of variation explained by the model relative to the mean. The closer the values of *R* to 1, the better is the correlation between the experimental and predicted responses (Sharma et al. 2009). Here, the value of *R* ^2^ (0.952) indicates good relation between the experimental and predicted values of the response. The predicted *R* ^2^ of 0.905 is in good agreement with the adjusted *R* ^2^ of 0.922, indicating that this RSM design can be used for modelling the design space. The linear factors of *X*_1_, *X*_2_ and *X*_3_; quadratic factors of *X*_1_, *X*_2_ and *X*_3;_ and interaction terms *X*_23_, *X*_24_ and *X*_34_ were found to be significant at 95 % confidence interval, indicating that the model terms are limiting factors for Congo red decolourization.

**Table 4:** ANOVA for the response surface quadratic model

Source of variation	Sum of squares	Degrees of freedom	Mean square	Ratio	Significance
Regression	2734.00	14	195.286	19.952	***
Residuals	137.03	14	9.788		
Validity	32.23	10	3.223	0.123	NS
Error	104.80	4	26.200		
Total	871.03	28			

*NS* nonsignificant

*** Significant at the level of 99.9 %

**Fig. 3:**
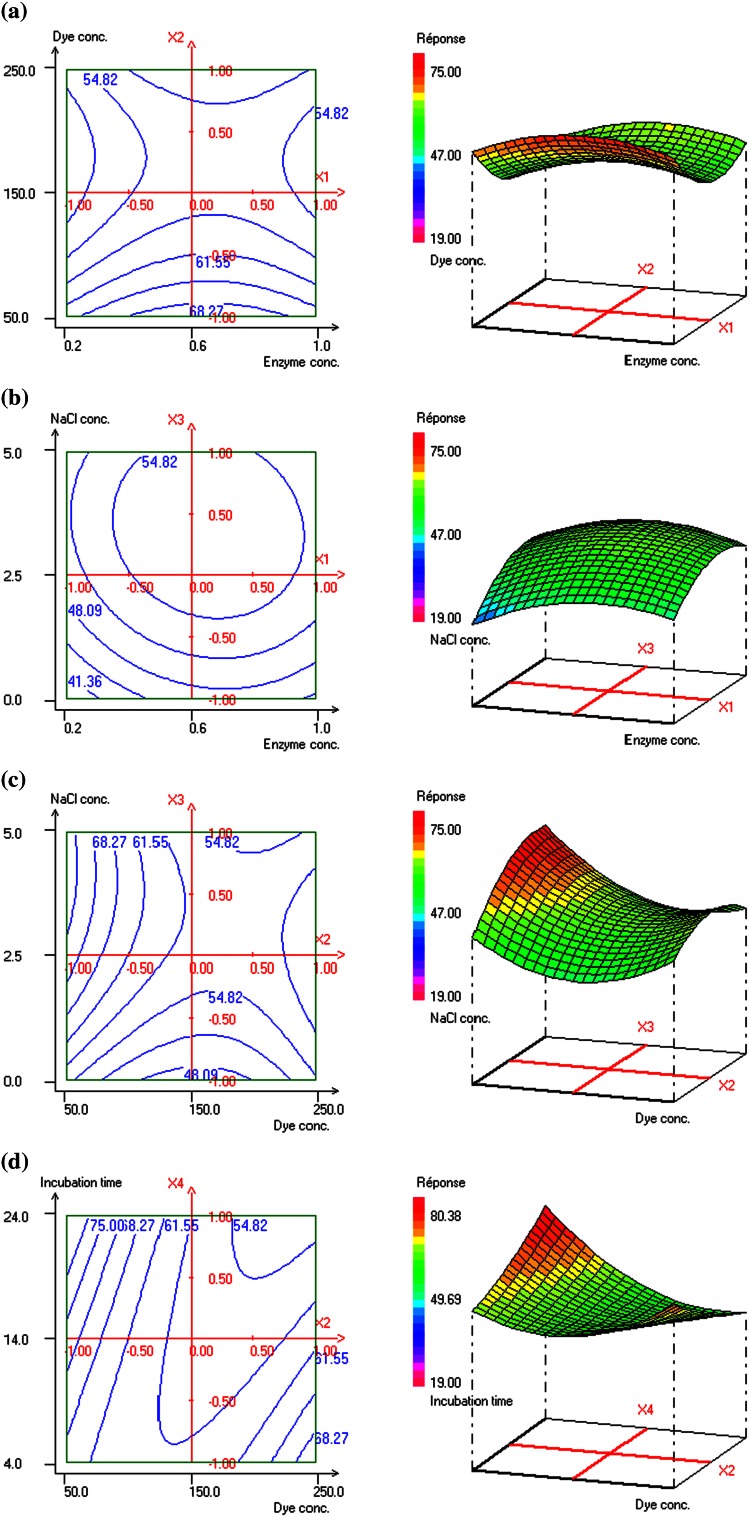
Contour and response surface plots of *Congo red* decolourization by *P.* *extremorientalis* laccase as a function of: **a** enzyme concentration (*X*_1_) and dye concentration (*X*_2_) levels at midlevel of NaCl concentration (2.5 %) and incubation time (14 h); **b** enzyme concentration (*X*_1_) and NaCl concentration (*X*_3_) levels at midlevel of dye concentration (150 mg/l) and incubation time (14 h); **c** dye concentration (*X*_2_) and NaCl concentration (*X*_3_) levels at midlevel of enzyme concentration (0.6 U/ml) and incubation time (14 h); **d** dye concentration (*X*_2_) and incubation time (*X*_4_) levels at midlevel of enzyme concentration (0.6 U/ml) and NaCl concentration (2.5 %)

Interactions between the studied variables for Congo red dye decolourization are shown in 3D and 2D contour plots (Fig. 3a–d). These plots show the Congo red decolourization as function of two factors, while the others were fixed at zero level. 3D and 2D contour plots for the interaction effect of enzyme and dye concentrations towards dye decolourization are shown in Fig. 3a. The results indicate that the response increased on increasing the enzyme concentration and decreasing the dye concentration. The decreasing dye decolourization at higher concentrations was probably a result of possible enzyme inactivation at such high dye levels. The behaviour of percentage decolourization with respect to changes in enzyme and salt concentrations is shown in Fig. 3b. These two parameters showed positive influence on dye decolourization. The percentage dye decolourization increased with increase in salt concentration and enzyme concentration until a certain level, where further increases in both parameters led to nonsignificant change in dye decolourization. Figure 3c represents the effect of varying NaCl and dye concentrations at fixed levels of enzyme concentration and incubation time. The response plot revealed that an increase in salt concentration increased the decolourization level. However, the rate of decolourization decreased with the increase in dye concentration. Figure 3d represents the effect of varying concentrations of dye at different incubation times on Congo red decolourization under 0.6 U/L enzyme and 1.1 mM salt concentrations. The results indicate globally that the response increased with the increase in the reaction time and decrease in the dye concentration and vice versa.

The optimum operating conditions, carried out numerically by using NemrodW software, are dye concentration 50 mg/l, enzyme concentration 0.6 U/ml, salt concentration 2.5–5 % and incubation time 24 h. The expected value of the Congo red decolourization yield was *y*_op_ = 81.5 ± 2.5 %. An additional experiment was carried out under the selected optimal decolourization conditions. It led to Congo red decolourization yield equal to 79.8 ± 2.1 %, which was in close agreement with the predicted response value. This result is in agreement with the study of Zhao et al. (2011) in which 70 % decolourization of Congo red was obtained in 24 h with spore-bound laccase from *B.* *sutilis* WD23. However, lower Congo red decolourization yields of about 42.86 and 36.09 % were registered at 96 h by partially purified laccases from *P.* *aeruginosa* and *P.* *fluorescens*, respectively (Peter and Vandana 2014; Vandana and Peter 2014). The most effective *Pseudomonas* laccases in the decolourization of textile dyes and effluents have been reported by Kalme et al. (2009) and Kuddus et al. (2013). The purified laccase from *P.* *desmolyticum* NCIM 2112 showed 100 % decolourization of Direct and reactive azo dyes, including Direct Blue-6, Green HE4B and Red HE7B (Kalme et al. 2009). The crude enzyme of *P.* *putida* MTCC 7525 showed about 36–94 and 16–86 % decolourization of synthetic dyes (20 mg/l) and industrial effluents (10 %), respectively, within 24 h of incubation (Kuddus et al. 2013).

## Conclusion

In the present study, a novel laccase enzyme-producing bacterium was selected and subjected to laccase production under solid-state fermentation conditions using wheat bran as a support substrate. The application of the enzyme to decolourize the recalcitrant azodye Congo red was investigated using experimental design and RSM. We concluded that *P.* *extremorientalis* laccase had great potential as biocatalyst in view of its activity and stability at alkaline pH, resistance to inhibition by halide ions as well as the ability to decolourize Congo red azo dye. Further studies, including enzyme purification, sequence determination, site-directed mutagenesis and crystallographic analysis of *P.* *extremorientalis* laccase, are required to elucidate more details about its stability against harsh conditions such as high salinity and alkalinity. A pilot-scale enzymatic decolourization study will be conducted with this valuable biocatalytic process for actual industrial applications.
